# Perinatal outcomes in twin late preterm pregnancies: results from an Italian area-based, prospective cohort study

**DOI:** 10.1186/s13052-022-01297-4

**Published:** 2022-06-16

**Authors:** Francesca Monari, Giuseppe Chiossi, Michela Ballarini, Daniela Menichini, Giancarlo Gargano, Alessandra Coscia, Dante Baronciani, Fabio Facchinetti, for the Late Preterm Emilia Romagna Group, Vittorio Basevi, Frusca Tiziana, Giuseppe Battagliarin, Marinella Lenzi, Gina Ancora, Luigi Corvaglia

**Affiliations:** 1 Obstetrics and Gynecology Unit, Mother-Infant and Adult Department of Medical and Surgical Sciences, University of Modena and Reggio Emilia, Policlinico di Modena, Via del Pozzo 71, 41125 Modena, Italy; 2 Department of Biomedical, Metabolic and Neural Sciences, International Doctorate School in Clinical and Experimental Medicine, University of Modena and Reggio Emilia, 41125 Modena, Italy; 3 Department of Obstetrics and Pediatrics, Neonatal Intensive Care Unit, Arcispedale Santa Maria Nuova, IRCCS, Reggio Emilia, 42123 Reggio Emilia, Italy; 4 Department of Public Health and Pediatrics, Neonatology Unit, Università degli Studi di Torino, 10126 Turin, Italy; 5 Health Facilities, Technologies and Information Systems Unit, Emilia-Romagna Region, Viale Aldo Moro 21, 40127 Bologna, Italy

**Keywords:** Twins, Monochorionic, Dichorionic, Late preterm, P PROM, Medical indication, Perinatal oucomes, Evidence based indication

## Abstract

**Background:**

Multiple gestations represent a considerable proportion of pregnancies delivering in the late preterm (LP) period. Only 30% of LP twins are due to spontaneous preterm labor and 70% are medically indicated; among this literature described that 16–50% of indicated LP twin deliveries are non-evidence based. As non-evidence-based delivery indications account for iatrogenic morbidity that could be prevented, the objective of our observational study is to investigate first neonatal outcomes of LP twin pregnancies according to gestational age at delivery, chorionicity and delivery indication, then non evidence-based delivery indications.

**Methods:**

Prospective cohort study among twins infants born between 34 + 0 and 36 + 6 weeks, in Emilia Romagna, Italy, during 2013–2015. The primary outcome was a composite of adverse perinatal outcomes.

**Results:**

Among 346 LP twins, 84 (23.4%) were monochorionic and 262 (75.7%) were dichorionic; spontaneous preterm labor accounted for 85 (24.6%) deliveries, preterm prelabor rupture of membranes for 66 (19.1%), evidence based indicated deliveries were 117 (33.8%), while non-evidence-based indications were 78 (22.5%).

When compared to spontaneous preterm labor or preterm prelabor rupture of membranes, pregnancies delivered due to maternal and/or fetal indications were associated with higher maternal age (*p* <  0.01), higher gestational age at delivery (*p* <  0.01), Caucasian race (p 0.04), ART use (*p* <  0.01), gestational diabetes (*p* <  0.01), vaginal bleeding (*p* <  0.01), antenatal corticosteroids (*p* <  0.01), diagnosis of fetal growth restriction (FGR) (*p* <  0.01), and monochorionic (*p* <  0.01). Two hundred twenty-six pregnancies (65.3%) had at least one fetus experiencing one composite of adverse perinatal outcome. Multivariate analysis confirmed that delivery indication did not affect the composite of adverse perinatal outcomes; the only characteristic that affect the outcome after controlling for confounding was gestational age at delivery (*p* <  0.01). Moreover, there was at least one adverse neonatal outcome for 94% of babies born at 34 weeks, for 73% of those born at 35 weeks and for 46% of those born at 36 weeks (*p* <  0.01).

**Conclusion:**

Our study suggests that the decision to deliver or not twins in LP period should consider gestational age at delivery as the main determinant infants’ prognosis. Delivery indications should be accurately considered, to avoid iatrogenic early birth responsible of preventable complications.

## Introduction

Late preterm (LP) birth, defined as delivery between 34 and 0/7 and 36–6/7 weeks of gestation, accounts for about 70% of overall prematurity in Europe [1]. Compared to the term neonates, LP more commonly encounter adverse outcomes, such as higher respiratory morbidity [2–4], long-term adverse developmental and cognitive outcomes [5] and may also have repercussions on their health as adults [6, 7].

Multiple gestations represent a considerable proportion of pregnancies delivering in the LP period. Twin gestations account for approximately 3–4% of all live births [8, 9], with a LP rate of 49.8% [10], and a mean gestational age (GA) at delivery of 35.3 weeks [11]. When compared to singleton gestations, twin mothers have higher morbidity and mortality rates from gestational hypertension, cesarean delivery, and postpartum hemorrhage, while their newborns have higher NICU admission rates and neonatal sequelae [12]. Furthermore, after adjusting for gestational age at delivery, monochorionic twins (MC) experience higher rates of perinatal complications and stillbirth (SB) than dichorionic twins (DT) and singletons [13–15]*.*

While 70% of all twin births are indicated, 30% are due to spontaneous preterm labor [16]. Despite guidelines indicating when to deliver twin gestations according to gestational age, chorionicity, and concomitant maternal or fetal complications [17], 16–50% of indicated LP twin deliveries are non-evidence based (EB) [17]. As non EB delivery indications account for iatrogenic morbidity that could be prevented, this observational study intended to investigate first neonatal outcomes of LP twin pregnancies according to GA at delivery, chorionicity and delivery indication, then non EB delivery indications.

## Materials and methods

We conducted a prospective, multicenter, area-based cohort study of the LP deliveries from December 1st 2013, to December 1st 2015 in Emilia Romagna, a region in Northern Italy with nearly 4.5 million people organized in 9 counties. The results of the single pregnancy cohort study were just published [18].

The study steering committee (consisting in 2 obstetricians, 2 neonatologists and 1 epidemiologist) defined the information to collect on pregnancies resulting in LP deliveries, and on neonates’ hospital course. Standardized chart abstraction forms were designed to obtain anonymized data on mothers (i.e maternal demographics, maternal medical and obstetrical complications, labor, and delivery details) and their newborns (i.e birthweight, gender, Apgar scores, admission to the NICU or Intermediate/Step Down Unit, and length of stay, neonatal morbidities, and mortality). In Emilia Romagna, inpatient obstetric and pediatric care was provided at the time of the study by 28 hospitals within the National Health System. Recruitment occurred only at the 21 hospitals with at least 500 deliveries/year, equipped with NICUs or Intermediate/Step Down Units, that could care for LP infants: one obstetrician and one neonatologist/pediatrician from each study site approached mothers with twin pregnancy delivering at 34^+ 0^–36^+ 6^ weeks for written informed consent, and respectively completed the maternal and the neonatal data collection forms, as they interviewed the patients and consulted the medical records. Five trained research associates visited the study sites monthly basis to review the maternal and neonatal data collection forms with the physicians that had filled them. At the Data Managing Center (Modena Policlinico Hospital, University of Modena, and Reggio Emilia) research associates used optical character recognition technology (Flexi Capture, Abbyy®, Germany) to scan the data collection sheets, extract pertinent data, and organize it in a password protected database. If automated data extraction failed (approximately 5% of the times), research associates reviewed the forms and manually entered the missing data in the database. Authors have complied with the World Medical Association Declaration of Helsinki regarding ethical conduct of research involving human subjects. Approval from the Institution Review Board of the 9 Emilia Romagna Counties was obtained (n 24,512 _ 25/9/2014). The study was financed by the Emilia Romagna County Grant (n 417,149 _ 2014).

### Study population

Women with a viable twin pregnancy were classified according to the indication for delivery as spontaneous preterm labor (sPTL), preterm prelabor rupture of membranes (pPROM) or indicated delivery. The diagnosis of preterm labor was based on clinical criteria of regular uterine contractions accompanied by progressive changes in cervical dilation and effacement [19]. As preterm labor occurred ≥34 weeks, tocolytic therapy was not administered. Women with DC pregnancy presenting with pPROM between 34^+ 0^- and 36 + 6-weeks’ gestation, who were not in labor within 24 hours after rupture of membranes and had no indication for immediate delivery, were expectantly managed as detailed in the PPROMEXIL trial [20]. Instead, MC pregnancies were not expectantly managed, because of the increased risks of placental abruption, twin to twin transfusion syndrome or intravascular acute shunting [21]. The diagnosis of pPROM was confirmed by visualization of amniotic fluid passing from the cervical canal and pooling in the vagina, a basic pH test of vaginal fluid, or arborization (ferning) of dried vaginal fluid, which is identified under microscopic evaluation. A course of therapy with a combination of a beta lactam and a macrolide antibiotic was left at the discretion of each study site even when pPROM occurred after 34 weeks, as a preliminary inquiry had found such practice to be common. Rupture of membranes was considered as the delivery indication, when spontaneous labor occurred more than 24 hours after pPROM among expectantly managed DC pregnancies, or also if the patient and/or her obstetrician opted to terminate expectant management with an elective delivery. If onset of labor occurred within 24 hours of rupture of membranes, sPTL was considered as the delivery indication; instead, if complications prompting delivery arose among expectantly managed pPROMs, such deliveries were classified as indicated.

Currently no consensus on whether RDS prophylaxis should be administered in twin pregnancies in the LP population, thus no specific recommendations were given concerning administration of antenatal corticosteroids (ANCS) in this period.

The timing of delivery was determined in completed weeks of gestation such that 34 weeks (for example) included deliveries at 34^+ 0^–34^+ 6^ weeks. Gestational age was based either on first trimester ultrasound scan or, in women with a regular cycle, on the first day of the last menstrual period if the expected date of delivery differed less than 7 days from that estimated by ultrasound.

Chorionicity was assigned in the 1st trimester of pregnancy by ultrasound assessment of the “twinpeak” or “lambda” sign [22]. After delivery, chorionicity was confirmed in all cases by inspection of the placenta and membranes, as well as by individual review of placental pathology, available in 80% of patients.

### Outcome measures

The primary outcome is a composite adverse neonatal outcomes, stratified by GA, chorionicity and delivery indication, including neonatal death, respiratory distress syndrome (RDS), transient tachypnea of the newborn (TTN), mechanical ventilation, hypoglycemia, newborn sepsis, confirmed seizures, stroke, intraventricular hemorrhage (IVH), cardiopulmonary resuscitation, umbilical-cord-blood arterial pH < 7.0 or base excess < − 12.5, a 5-minute Apgar score ≤ 3, NICU length of stay ≥5 days. As indicated by previous studies, these outcomes are associated with significant risks of neonatal mortality or long-standing adverse health complications, including hypoxic ischemic encephalopathy [23]. Neonatal ipoglicemia was defined as low blood glucose level < 40 mg/dl before 48 hours age [24]. Intraventricular hemorrhage was defined as the presence of blood within the ventricular system including the lateral, third and fourth ventricles. The assessment of IVH grade was based on the Papile’s classification [25]: 1) haemorrhage restricted to the subependymal germinal matrix; 2) subependymal and intraventricular haemorrhage, without ventricular dilatation; 3) subependymal germinal matrix haemorrhage, intraventricular haemorrhage, and ventricular dilatation; and 4) parenchymal haemorrhage associated with intraventricular dilatation.

Non reassuring fetal status was defined as category III [26] or persistent category II fetal heart rate pattern with abnormal labor progress [27], non-reactive non stress test (NST) associated with recurrent decelerations among non-laboring women [28], absent or reverse umbilical artery end diastolic flow in the setting of fetal growth restriction (FGR) [29]. Clinical chorioamnionitis was defined as Triple I [30] with the presence of fever without a clear source plus any of the following: 1) baseline fetal tachycardia (greater than 160 beats per min for 10 min or longer, excluding accelerations, decelerations, and periods of marked variability), 2) maternal white blood cell count greater than 15,000 per mm^3^ in the absence of corticosteroids and 3) definite purulent fluid from the cervical os.

CPAP and oxygen administration represented non-invasive respiratory support, as opposed to mechanical ventilation. Neonatal sepsis was defined as a clinical syndrome prompting antibiotic treatment, with or without positive cultures [31]. Cerebral lesions were suspected clinically, screened by neonatal brain ultrasound, and confirmed on MRI [3].

Independent medical records reviews by 2 senior authors determined if LP deliveries were due to sPTL, pPROM, maternal and/or fetal indications. Reviewers also determined if indicated deliveries were EB or not EB, according to ACOG [32], as well as Italian guidelines [33]. Discordance between reviewers was resolved consulting a 3rd author. When multiple indications were listed, deliveries were due to EB indications if at least one of them was consistent with the adopted guidelines. The following were considered EB LP twin delivery indications: elective CS in MC twin, preeclampsia with severe features/HELLP syndrome/eclampsia, pPROM in MC twins, FGR with abnormal antenatal testing (abnormal testing included a biophysical profile of 6/10 or worse, abnormal umbilical artery or ductus venosus Doppler, or coexisting oligohydramnios), vaginal bleeding due to placental abruption, non-reassuring fetal heart tracing (a category III fetal heart tracing requiring immediate delivery) [34], twin to twin transfusion syndrome stage II ore above [35], clinical chorioamnionitis [30], and pre-gestational diabetes mellitus not under metabolic control. Deliveries defined as non EB included stable patients such as those with mild chronic hypertension [18], prior myomectomy, prior classical cesarean delivery, or mild cholestasis [36] of pregnancy.

### Statistical analysis

As we stratified neonatal outcomes according to delivery indications, we also compared how maternal, fetal, and neonatal characteristics varied in case of sPTL, pPROM or deliveries prompted by maternal and/or fetal conditions. Categorical variables were presented as n (%) and tested with Chi square test or Fisher’s exact test as appropriate. Normally distributed continuous variables were presented as mean ± SD and compared with One Way ANOVA. Non-normally distributed continuous variables were presented as median (IQR) and tested with One Way ANOVA on ranks. A level of statistical significance of *P* ≤ 0.05 was considered.

Multivariate logistic regression analysis was used to investigate if delivery indications independently affected the risk of adverse neonatal outcomes. The following variables were tested as potential confounders: maternal age, parity, race, low education, smoking, maternal BMI, excessive weight gain, utilization of assisted reproductive technologies (ART), treatment with antenatal corticosteroids (ANCS), ASA, low molecular weight heparin (LMWH), or progesterone.

The strength of the association between the covariates and the dependent variable was estimated as area under the curve of a receiver operating characteristic (ROC) curve plotted with the true-positive rate compared with the false positive rate. Statistical analyses were performed using Stata 15 (StataCorp, College Station, TX).

## Results

Among the 346 LP twin gestations included in our cohort, 84 (23.4%) were monochorionic pregnancies (of which only 3 were MCMA), while 262 (75.7%) were dichorionic; sPTL accounted for 85 (24.6%) deliveries, pPROM for 66 (19.1%), EB indicated deliveries were 117 (33.8%), while non EB indications were 78 (22.5%).

In the univariate analysis shown in Table 1, maternal and obstetrics characteristics are presented according to delivery indication. When compared to sPTL or pPROM, pregnancies delivered due to maternal and/or fetal indications were associated with higher maternal age (*p* <  0.01), higher gestational age at delivery (*p* <  0.01), Caucasian race (p 0.04), ART use (*p* <  0.01), gestational diabetes (*p* <  0.01), vaginal bleeding (*p* <  0.01), prescription of antenatal corticosteroids (*p* <  0.01), diagnosis of FGR (*p* <  0.01), and were more frequently monochorionic (*p* <  0.01). Only 15.3% (53/546) of pregnancies had a vaginal delivery of both fetuses. Cesarean deliveries in non-laboring women were 204 (58.9%), induced labor 37 (10.7%), while sPTL were 105 (30.4%). Women experiencing sPTL had the highest rate of vaginal delivery (27.1%).

**Table 1:** Maternal and fetal characteristics of twins according to delivery indications

	Spontaneous n 85 (24.6%)	P PROM n 66 (19.1%)	Indicated n: 117 (33.8%)	No indicated n: 78 (22.5%)	Total n: 346	***P***
**Mean maternal age**	32 ± 5.6	33.8 ± 5.9	34.7 ± 5.7	34.6 ± 5.8	33.9 ± 5.8	< 0.01+
**Primiparity**	38 (44.7)	27 (40.9)	51 (43.6)	41 (52.6)	157 (45.4)	0.5*
**Chorionicity**						
*Bichorial-biamniotic*	66 (77.6)	57 (86.4)	61 (52.1)	78 (100)	262 (75.7)	< 0.01**
*Monochorial-biamniotic*	19 (22.4)	9 (13.6)	53 (45.3)	0	81 (23.4)	
*Monochorial-monoamniotic*	0	0	3 (6)	0	3 (0.9)	
**Previous spontaneous PTB**	5 (5.9)	0	1 (0.8)	3 (3.8)	9 (2.6)	0.06**
**Gestational age**						
*34–34 + 6 weeks*	23 (27.1)	14 (21.2)	24 (20.5)	12 (15.4)	73 (21.1)	< 0.01*
*35–35 + 6 weeks*	33 (38.8)	26 (39.4)	35 (29.9)	16 (20.5)	110 (31.8)	
*36–36 + 6 weeks*	29 (34.1)	26 (39.4)	58 (49.6)	50 (64.1)	163 (47.1)	
**Race**						
*Non caucasian*	26 (30.6)	12 (18.2)	23 (19.7)	10 (12.8)	71 (20.5)	0.04*
**Low education (< 8 years)**	25 (29.4)	18 (27.3)	27 (23.1)	13(16.7)	83 (24)	0.2*
**BMI**	22.1 (20.2–27.3)	22.7 (20.2–26.9)	22.9 (20.3–25.8)	21.8 (19.8–25.7)	22.4 (20.2–26.2)	0.6^§^
**Obesity**	14 (16.9)	12 (18.7)	16 (14)	7 (9)	49 (14.4)	0.3*
**Excessive Weight Gain (IOM)**	10 (17.5)	6 (15)	13 (16.2)	4 (6.8)	33 (14)	0.3**
**Smoking habit**						
*Smoking*	6 (7.2)	9 (13.6)	9 (8)	12 (15.4)	36 (10.6)	0.2*
**Assisted reproductive technologies**	18 (21.2)	12 (18.2)	30 (25.6)	32 (41)	92 (26.6)	< 0.01*
**Diabetes**						
*Pregestational diabetes*	0	0	1 (0.8)	0	1 (0.3)	< 0.09**
*Class A2 GDM*	1 (1.2)	3 (4.5)	7 (6)	0	11 (3.2)	
*Class A1 GDM*	6 (7)	2 (3)	12 (10.3)	4 (5.1)	24 (6.9)	
*No diabetes*	78 (91.8)	61 (92.5)	97 (82.9)	74 (94.9)	310 (89.6)	
**Hypertensive disorders**						
*Chronic Hypertension*	0	0	1 (0.9)	2(2.6)	3 (0.9)	< 0.01 **
*Preeclampsia/ Gestational*	0	5 (7.6)	33 (28.2)	0	38 (11)	
*Hypertension*	85 (100)	61 (92.4)	83 (70.9)	76 (97.4)	305 (88.1)	
*Normotensive*						
**Vaginal bleeding (Abruption/Placenta previa)**	1 (1.2)	2 (3)	13 (11)	0	16 (4.6)	< 0.01**
**Liver disorders**	5 (5.9)	6 (9.1)	4 (3.4)	5 (6.4)	20 (5.8)	0.04 **
**Twin to twin transfusion syndrome**	0	0	3 (2.4)	0	3 (0.9)	0.3**
**Clinical chorioamnionitis**	4 (4.7)	2 (3)	0	0	6 (1.7)	0.01**
**Type of labor**						
*No labour*	0	41 (62.1)	97 (82.9)	66 (84.6)	204 (58.9)	< 0.01**
*Induced labour*	0	5 (7.6)	20 (17.1)	12 (15.4)	37 (10.7)	
*Spontaneous labour*	85 (100)	20 (30.3)	0	0	105 (30.4)	
**Mode of delivery**						
*Vaginal*	23 (27.1)	11 (16.7)	12 (10.3)	7 (9)	53 (15.3)	< 0.01**
*Cesarean in labour*	62 (72.9)	14 (21.2)	8 (6.8)	5 (6.4)	89 (26)	
*Cesarean non in labour*	0	41 (62.1)	97 (82.9)	66 (84.6)	204 (58.7)	
**Antenatal corticosteroids**						
*No steroids*	50 (58.8)	29 (43.9)	91 (77.8)	45 (57.7)	215 (62.1)	< 0.01*
**ASA prophylaxis**	5 (5.9)	4 (6.1)	6 (5.1)	4 (5.1)	19 (5.5)	0.9**
**LMWH prophylaxis**	4 (4.4)	3 (4.5)	3 (2.6)	3 (3.8)	13 (3.8)	0.8**
**Progesterone Prophylaxis**	10 (15.3)	4 (6.1)	13 (11.1)	10 (14.8)	40 (11.6)	0.3**
**Non reassuring fetal monitoring (category III)**	2 (2.3)	2 (3)	5 (4.3)	0	9 (2.6)	0.3**
**Fetal Growth restriction (FGR)**	3 (3.5)	0	30 (25.6)	0	33 (9.5)	< 0,01**
**Fetal anomalies**						
*None*	82 (96.5)	66 (100)	112 (95.7)	78	338 (97.7)	0.8**
*Cardiovascular*	2 (2.3)	0	3 (2.5)	0	5 (1.4)	
*CNS*	1 (1.2)	0	0	0	1 (0.3)	
*Gastrointestinal*	0	0	1 (0.9)	0	1 (0.3)	
*Genito-urinary*	0	0	1 (0.9)	0	1 (0.3)	
**Amniotic fluid anomalies**						
*Oligo/anidramnios*	1 (1.2)	0	2 (1.8)	0	3 (0.9)	0.8**
*Polidramnios*	1 (1.2)	0	1 (0.9)	0	2 (0.6)	
*Normal*	83 (97.6)	66 (100)	114 (97.3)	78 (100)	341 (98.5)	

Low education: primary and secondary school; Excessive Weight Gain: above IOM guidelines per BMI category; Previous PTB: prior birth < 37 weeks; Class A1 GDM: diet, Class A2 GDM: Insulin therapy; Non reassuring fetal monitoring: category III tracing according to ACOG

^*^Chi square test

^**^ Fisher Exact test

^§^ANOVA on ranks, + ANOVA

Table 2 summarizes neonatal characteristics. Two hundred twenty-six pregnancies (65.3%) had at least one fetus experiencing one adverse outcome; pregnancies with sPTL, pPROM and indicated deliveries had similar rates of the composite adverse neonatal outcome. Newborn characteristics were similar among study groups; however, mothers with indicated deliveries had lower birthweights (*p* <  0.01) and higher SGA rates (*p* <  0.01). Median NICU length of stay was similar among study groups, although stay ≥5 days was less common when indicated deliveries were non EB (*p* <  0.05).

**Table 2:** Neonatal outcomes in twins according to delivery indications

	Spontaneous n: 165	pPROM n: 128	Indicated n: 232	No indicated n: 152	Total (n 677)	***P***
**Male**	92 (55.8)	52 (40.6)	120 (51.7)	71 (46.7)	335 (49.5)	0.06*
**Mean birth weight**	2352 (±334)	2355 (±383)	2257 (±359)	2349 (±375)	2319 (±363)	**0.6**^**§**^
**Stillbirth**	1 (0.6)	1 (0.8)	4 (1.6)	1 (0.7)	7 (1%)	0.8**
**Weight percentile**						
*AGA*	146 (88.5)	112 (87.5)	174 (75)	121 (79.6)	553 (81.7)	< 0.01**
*LGA*	3 (1.8)	3 (2.3)	2 (0.9)	4 (2.6)	12 (1.8)	
*SGA*	16 (9.7)	13 (10.3)	56 (24.1)	27 (17.8)	112 (16.5)	
**Median NICU length of stay**	6 (3–8)	5 (3–8)	5 (3–10)	4 (3–7)	5 (3–8)	**< 0.06**^**+**^
**NICU stay longer than 5 days**	101 (61.2)	73 (57)	140 (60.3.)	72 (47.4)	386 (57)	< 0.05*
**Metabolic acidosis at birth**	8 (4.8)	5 (3.9)	6 (2.4)	0	19 (2.8)	0.02**
**5′ Apgar score < 3**	0	1 (0.8)	0	0	1 (0.1)	0.2**
**Cardiopulmonary resuscitation**	8 (4.8)	5 (3.9)	6 (2.4)	0	19 (2.8)	0.02**
**Respiratory support**						
*No support*	140 (84.8)	108 (84.4)	192 (82.8)	131 (86.2)	571 (84.3)	0.7**
*Invasive*	3 (1.8)	1 (0.8)	2 (0.8)	0	6 (0.9)	
*Non invasive*	22 (13.4)	19 (14.8)	38 (16.4)	21 (13.8)	100 (14.8)	
**Seizures**	0	1 (0.8)	0	0	1 (0.1)	0.2**
**Therapeutic hypothermia**	0	1 (0.8)	0	0	1 (0.1)	0.2**
**Jaundice**	38 (23)	21 (16)	40 (17.2)	21 (13.8)	120 (17.3)	0.2*
**Sepsis**	7 (4.2)	8 (6.2)	5 (2)	2 (1.3)	22 (3.2)	0.08**
**Antibiotic therapy**	10 (6.1)	12 (9.4)	10 (4)	3 (2.2)	35 (5.2)	0.04**
**Hypoglycemia**	41 (25.1)	30 (23.4)	57 (24.7)	35 (23.8)	163 (24.4)	0.9*
**Difficulty feeding**	17 (10.3)	23 (18)	32 (13.8)	17 (11.2)	89 (13.1)	0.2*
**Cerebral lesions**						
*None*	157 (95.1)	122 (95.3)	229 (98.7)	151 (99.3)	659 (97.3)	< 0.01**
*Stroke*	0	0	0	0	0	
*Basal nuclei anomalies*	8 (4.9)	6 (4.7)	3 (1.3)	0	17 (2.5)	
*IVH > =2*	0	0	0	1 (0.7)	1 (0.2)	
**Congenital anomalies**						
None	152 (92.1)	123 (96.1)	214 (92.2)	144 (94.8)	633 (93.5)	0.5**
*Cardio vascular*	7 (4.2)	5 (3.9)	11 (4.7)	6 (4)	29 (4.3)	
*CNS*	1 (0.7)	0	0 0	1 (0.6)	2 (0.3)	
*Gastrointestinal*	0	0	2 (0.9)	0	2 (0.3)	
*Genito-urinary*	5 (3)	0	5 (2.2)	1 (0.6)	11 (1.6)	
**Pregnancies with at least one infant affected by the Adverse Composite Neonatal Outcome**	58 (72.5)	43 (68.2)	79 (70.5)	46 (62.2)	226 (68.7)	0.5*

Matabolic acidosis: umbilical-cord-blood arterial pH < 7.0 or base excess < −12

^*^Chi square test

^**^Fisher Exact test

^§^ANOVA on ranks, + ANOVA

Table 3 lists the EB indicated delivery (117, 33.8%) and non EB indicated deliveries (78, 22.5%) according to our expert review.

**Table 3:** EB and NEB indicated delivery

EB Indicated deliveries n: 117 (33.8%)		NEB indicated deliveries n 78 (22.5%)	
**Fetal indication**		**Fetal indication**	
*Non reassuring fetal monitoring (category III)*	***7 (5.9)***	*Suspected fetal monitoring (category II)*	*5 (6.4)*
*Fetal Growth restriction (FGR)*	***25 (21.4)***	*Fetal Growth restriction (FGR) with normal testing*	*10 (12.9)*
*Monochorionic*	***46 (39.3)***	*Not specified*	*16 (20.5)*
*Fetal anomalies*	***2 (1.6)***		
*Other (cord prolapse)*	***1 (0.9)***		
**Maternal indication**		**Maternal indication**	
		*Prior myomectomy/ crsarean section*	*4 (5.1)*
*Gestational Hypertension/preeclampsia*	***21 (18)***	*Mild Gestational Hypertension*	*10 (12.8)*
*Pregestational Diabetets*	***1 (0.9)***	*Gestational Diabetets in diet therapy*	*15 (19.2)*
*Vaginal bleeding (Abruption/Placenta previa)*	***12 (10.3)***	*Mild cholestasis*	*3 (3.8)*
*Twin to twin transfusion syndrome*	***2 (1.7)***	*Mild chorinc hypertension*	*2 (2.6)*
*Clinical chorioamnionitis*	***0***	*Not specified*	*13 (16.7)*

As detailed in Table 4, multivariate analysis confirmed that delivery indication did not affect the composite adverse neonatal outcomes; the only characteristic that continued to affect the outcome after controlling for confounding was gestational age at delivery (*p* <  0.01). Moreover, there was at least one adverse neonatal outcome for 94% of babies born at 34 weeks, for 73% of those born at 35 weeks and for 46% of those born at 36 weeks, with a significant difference (*p* <  0.01).

**Table 4:** Multivariate logistic regression models investigating the role of gestational age, chorionicity, order and circumstances at delivery on neonatal outcomes

	Composite adverse neonatal outcomes	
	AOR (95%CI)	***p***
**Gestational age**		
*34*	**20.2 (6.9–58.3)**	**< 0.01**
*35*	**3.3 (1.9–5.6)**	**< 0.01**
*36*	§	
**Delivery indications**		
*Spontaneous PTL*	§	
*pPROM*	0.98 (0.46–2)	0.9
*Indicated*	1.36 (0.7–2.6)	0.9
*No indicated*	1.02 (0.5–2.1)	0.3

The following variables were tested as potential confounders: maternal age, parity, previous preterm birth, race, education, BMI, excessive wait gain, smoking, utilization of assisted reproductive technologies, treatment with LDA, progesterone, or LMWH, antenatal corticosteroids

The area under receiver operating characteristic (ROC) curve was respectively 0.67 for composite adverse neonatal outcomes, 0.67 for neonatal resuscitation, 0.66 for metabolic complications, and 0.69 for respiratory support

*AOR* adjusted OR

## Discussion

Although outcomes of singleton gestations at 34^+ 0^–36^+ 6^ were shown to vary according to delivery indications [37], our large prospective cohort of twin gestation did not corroborate such finding. Instead, we confirmed on twins that significant differences in neonatal outcomes depend on gestational age at delivery, as it was previously established for singletons [38]. Furthermore, we showed that almost 1 out of 4 twin gestation in our cohort was delivered due non EB indications.

The high prevalence of non EB delivery indications in our twin population raises great concerns. Twin pregnancies represent a fragile obstetric population, having worse outcomes than singletons [38]; furthermore, LP deliveries are associated with worse outcomes than early term and term deliveries [10, 39]. Therefore, lack of sound indications for twin deliveries in the LP window combines the downsides of prematurity with the ones of multiple gestations, multiplying the risks of severe complications. Our findings urge a critical review of clinical practice encouraging obstetric providers to familiarize with the indications that prompt delivery among twins, overcome the uneasiness associated with management of twin pregnancies, in order to prevent iatrogenic morbidity. In our cohort 10 deliveries (12.8%) were due to gestational hypertension, 15 (19.2%) to class A1 gestational diabetes mellitus (diet therapy), 2 (2.6%) to mild cholestasis of pregnancy, while no clear indication was stated in 29 instances (37.2%) and translate into 78 (22.5%) early deliveries that could be avoided.

Our results are consistent with previous reports on singleton gestations [40] showing higher rates of non-indicated deliveries in the LP window. Moreover, Reddy et al. reviewed delivery indications for almost 3.5 million births in the United States concluding that 23% of LP births had no documented indication: the author speculated that many of these births without listed indications were due to perceptions of impending morbidity and mortality risks by either patients or providers [41].

The balance between the downsides of prematurity and the risks associated with pregnancy continuation, can be hard to strike in the LP period, especially among twin gestations [31] accounting for a discrepancy in clinical practice according to hospital settings and countries [42]. A recent Delphy survey on delivery indications in singleton pregnancies, showed that Italian experts generally agreed to be more conservative at 34 weeks, favoring delivery at 36 weeks, whatever the clinical scenery [32]. Nevertheless, in our twin population the most non EB indicated deliveries (22.5%) were without a clear medical cause, maybe due to different clinical setting and provider advisability.

Although women with EB deliveries were more likely to have babies with adverse perinatal outcome, this did not remain significant in the multivariate analysis, where the only factor affecting neonatal morbidity was the gestational age at delivery, as demonstrated by other studies [43]. Delivery indication did not significantly affect the outcome of interest even after excluding from the analysis non EB indicated delivery (data not shown).

Six stillbirths were detected in our population: 5 among BC pregnancies, 1 among MC twins. Our stillbirth rate (1.7%) is consistent with the current literature; however, we did not find any difference according to chorionicity [15]. Furthermore, chorionicity did not affect the composite adverse neonatal outcome as shown in the multivariate analysis. Previous reports have shown greater neonatal morbidity and mortality among MC pregnancies, from placental vascular complications [44] such as in TTTS, or in case of intravascular shunting after a single intrauterine death; or due to congenital malformations, selective growth restriction, or maternal complications [13, 14, 45]. We can speculate that improved outcomes among MC twins could be attributed not only to an evidence based antenatal care [12, 46] but also to a generally healthier obstetric population, where risk factors such as obesity [47], hypertensive disorders and diabetes have relatively low prevalence.

The joined 2020 NIH [48] and SMFM consensus recommended delivery at 38 weeks’ gestation for uncomplicated DC twin pregnancies as opposed to 34–37 weeks’ gestation for uncomplicated MC twins [12]. This broad interval was recently revised by a retrospective cohort study showing that elective LP delivery of MC is associated with significantly increased neonatal morbidity that does not seem justifiable by a corresponding reduction in the risk of SB. Therefore, the authors concluded that with a specialized antenatal care for twins and intensive fetal surveillance, delivery at 37 weeks’ gestation appears to be associated with the best outcomes for uncomplicated MC [13]. Our data confirm such findings, suggesting to avoid LP birth both in uncomplicated MC and DC twins to reduce unnecessary admission to NICU and other neonatal complications.

Strengths of this study include the large sample size, area based and multicenter nature of the cohort: both characteristics increase the generalizability of our findings. The prospective design of the survey, along with predefinition of standardized chart abstraction forms completed by obstetricians and pediatricians, and periodically audited by research associates, limit misclassification bias and assures data validity. We also acknowledge some limitations. As the study did not provide clinicians with prespecified delivery indications, it could be challenging at times for study personnel to identify the rationale behind an indicated delivery. However, such lack of standardization also allowed us to collect information about indications that were non EB. Although inconsistencies in ANCS administration may reflect different opinion leaders’ viewpoints, we considered betamethasone treatment among the potential confounders in our multivariate analyses. Moreover, our data are related to the years 2013–2015 and clinical practices might have slightly changed in these years. Finally, due to the exploratory nature of our study, a specific sample size calculation was not performed.

## Conclusion

To our knowledge, this is one of the largest prospective studies investigating delivery indications among twin gestations, suggesting that the decision to deliver or not twins in the LP period should consider gestational age at delivery as the main determinant infants’ prognosis. Delivery indications should therefore be accurately considered, to avoid iatrogenic early deliveries responsible of preventable complications. Our findings can also be helpful when counselling mothers at risk of LP delivery and can be used to plan interventions for their newborns.

## Data Availability

The datasets generated during and/or analyzed during the current study are available from the corresponding author on reasonable request.

## Abbreviation

LP: Late preterm
FGR: Fetal growth restriction
p PROM: Preterm prelabor rupture of membranes
GA: Gestational age
NICU: Neonatal intensive care unit
MC: Monochorionic
DC: Dichorionic
EB: Evidence based
sPTL: Spontaneous preterm labor
RDS: Respiratory distress syndrome
TTN: Transient tachypnea of the newborn
IVH: Intraventricular hemorrhage
NST: Non stress test
CPAP: Continuous Positive Airway Pressure
HELLP: Hemolysis, elevated liver enzymes, low platelet count
ART: Assisted reproductive technologies
BMI: Body mass index
TTTS: Twin-to-twin transfusion syndrome
NIH: National institute of health
SMFM: Society of maternal fetal medicine
